# Successful Treatment of Chronic Disseminated Candidiasis Complicated by Immune Reconstitution Inflammatory Syndrome in a Child With Acute Lymphocytic Leukemia

**DOI:** 10.7759/cureus.44103

**Published:** 2023-08-25

**Authors:** Sultan Mosleh, Dima Rabaia, Thabet Zidan

**Affiliations:** 1 Pediatrics Hematology and Oncology, An-Najah National University Hospital, Nablus, PSE; 2 Faculty of Medicine and Health Sciences, An-Najah National University, Nablus, PSE

**Keywords:** invasive fungal infection, hepatosplenic candidiasis, acute lymphoblastic leukemia, corticosteroids, immune reconstitution inflammatory syndrome, chronic disseminated candidiasis

## Abstract

Chronic disseminated candidiasis (CDC) is a severe form of disseminated fungal infection that commonly affects the liver, spleen, and kidneys. In rare cases, CDC can be further complicated by an excessive immune response known as immune reconstitution inflammatory syndrome (IRIS). This syndrome occurs during the phase of immune recovery and is characterized by a systemic inflammatory response and excessive release of cytokines.

We present a case of a two-year-old female with a medical history of acute lymphocytic leukemia on chemotherapy. She was admitted with high fever refractory to conservative management that included broad-spectrum antimicrobials. Additionally, multiple skin lesions and a left-sided limp were noted. Whole-body imaging revealed multiple abscesses in the spleen, kidneys, scalp, and left lower limb. The culture of an aspirate material from skin lesions grew *Candida tropicalis. *Despite receiving appropriate antifungals, the patient showed no signs of improvement, leading to the diagnosis of CDC-induced IRIS. The patient was started on systemic corticosteroids, which resulted in rapid improvement in the patient’s clinical status, resolution of fever, and significant reduction in inflammatory markers.

## Introduction

Chronic disseminated candidiasis (CDC) is a severe form of disseminated fungal infection, commonly affecting liver, spleen, and less commonly, kidneys [1]. This condition poses a great risk to patients with weak immunity, such as those with neutropenia and malignancies. Based on the available literature, this condition is known to affect less than 5% of patients with acute leukemia [2]. In rare cases, CDC can be complicated by an inflammatory systemic response and cytokines storm known as immune reconstitution inflammatory syndrome (IRIS). This syndrome is characterized by a dysregulated immune response that occurs in the phase of neutrophil recovery, causing clinical deterioration of a new onset (unmasking) or a previously diagnosed (paradoxical) infection [3]. 

To the best of our knowledge, very few cases have been reported that highlight the effectiveness of corticosteroids used as an adjuvant therapy to treat CDC-induced IRIS in patients with hematologic malignancy [4]. Here, we report a two-year-old female patient with acute lymphocytic leukemia (ALL), who developed CDC-induced IRIS, and was successfully treated with long term antifungal therapy and corticosteroids.

## Case presentation

A two-year-old female patient who had been diagnosed with B-cell ALL was being treated according to St. Jude Total 16 (TOTXVI) therapy protocol (6-mercaptopurin, vincristine, and dexamethasone). During her fourth week of remission induction, her parents brought her to the emergency department with the complaint of a fever of 38.5 C for the past 24 hours. The parents mentioned that the fever was partially relieved by cold compressors at home. They denied any chills, sweats, cough, nausea, vomiting, or changes in bowel movement. On admission, she appeared well and active and exhibited stable vital signs. Additionally, her physical examination was unremarkable with no rash or skin lesions. 

Laboratory tests showed a leukocyte count of 1.5*10^3^/mm^3^ (normal range 4-15.5 *10^3^ cells/mm^3^), platelet count of 41*10^3^/mm^3^ (normal range 150-450*103/mm3), an absolute neutrophil count (ANC) of 280/mm^3^ (normal range 1.5-8.5*10^3^/mm^3^), and a C-reactive protein (CRP) of 66.72 mg/dl (normal < 0.8 mg/dl). Kidney and liver function tests, electrolytes, and uric acid were all within normal ranges. A full septic workup was done for complete blood count (CBC), blood culture, urinalysis and culture, cerebrospinal fluid analysis and culture, and chest x-ray. She was started on an empiric antimicrobial therapy consisting of intravenous amikacin, intravenous ceftazidime, oral acyclovir, and intravenous fluconazole. The septic workup results were all negative, and she continued to have a high fever without any signs of recovery. Five days later, the patient developed painless subcutaneous nodules, and she refused to walk. On skin examination, non-tender, brownish skin nodules were noted on the upper and lower limbs, measuring 0.5 x 0.5 cm, with the largest nodule measuring 1 x 1 cm. Lower limb examination was significant for hypotonia in the right lower limb with absent deep tendon reflexes. 

**Figure 1:**
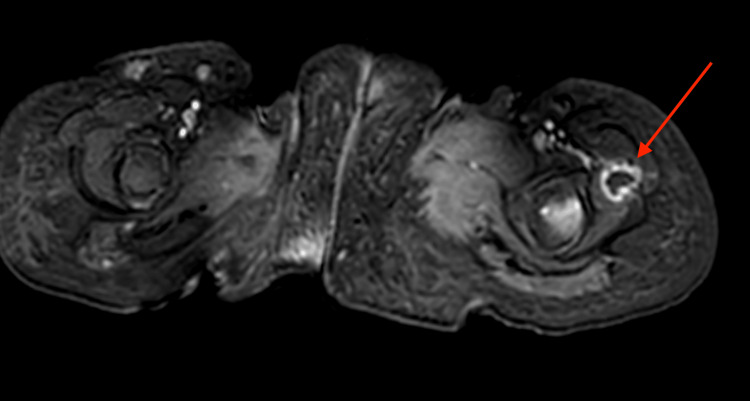
MRI of the hip Transverse view hip MRI showing an abscess (red arrow) within the vastus lateralis muscle adjacent to the proximal left femoral metaphyseal-diaphyseal junction.

**Figure 2:**
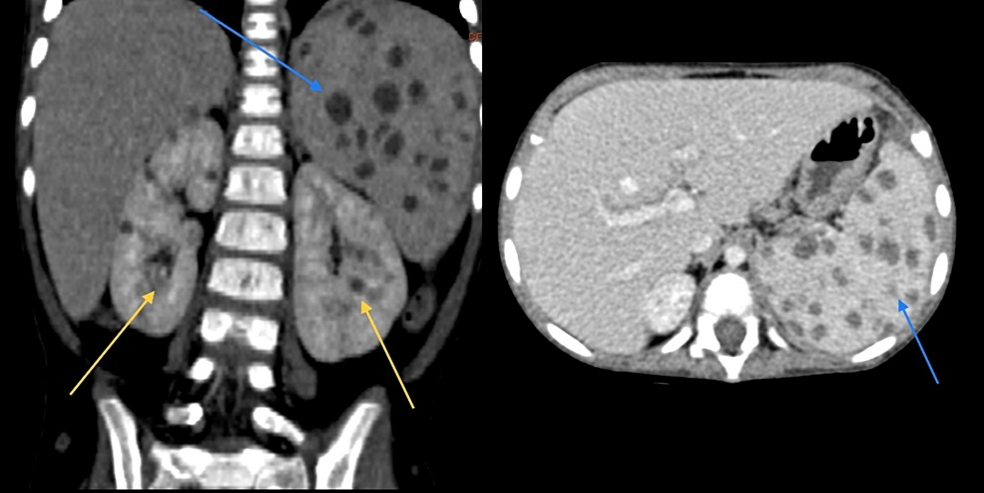
CT scan of abdomen Abdominal CT scan showing liver enlargement and multiple abscesses in the spleen (blue arrows) and kidneys (yellow arrows).

Chest x-ray and echocardiography revealed no pathological findings. A hip magnetic resonance imaging (MRI) revealed multiple abscesses proximal to the femoral metaphyseal-diaphyseal junction, with the largest abscess measuring 1.2 x 1.6 cm (Figure 1). Six days after admission, laboratory tests showed an ANC of 4290/mm^3^ (normal range 1.5-8.5*10^3^/mm^3^) and a leukocyte count of 6.98*10^3^/mm^3^ (normal range 4-15.5*10^3^ cells/mm^3^). A culture of an aspirate material obtained from a left cubital fossa abscess confirmed the presence of *Candida tropicalis*, which was sensitive to fluconazole. Over the following days, the patient continued to have high-grade fever with tenderness and an increased number of subcutaneous lesions. On examination, enlarged cervical and axillary lymph nodes were also noted. A repeat CRP test was 117.43 mg/dl (normal < 0.8 mg/dl). Given the disseminated and metastatic nature of the infection, she was started on IV amphotericin-B. Full body imaging showed an enlarged liver and spleen, along with multiple abscesses in the spleen, kidneys, scalp, abdominal wall, and gluteus muscles (Figure 2). These findings indicated the presence of splenic CDC. 

Despite receiving the appropriate antifungal therapy, no clinical improvement was observed in the patient’s condition. The patient’s condition continued to deteriorate, and her CRP remained elevated at 102.1 mg/dl (normal < 0.8 mg/dl). Given the paradoxical clinical worsening, characterized by disseminated soft tissue abscesses and a cytokine storm after the initiation of chemotherapy, and within the context of an underlying candida infection, she was diagnosed with IRIS, a condition triggered by immune recovery leading to systemic inflammation and a cytokine storm. Hence, the portal catheter was urgently removed, oral prednisolone (5 mg twice daily) was added, and amphotericin-B was switched to IV caspofungin as appropriate. 

**Figure 3:**
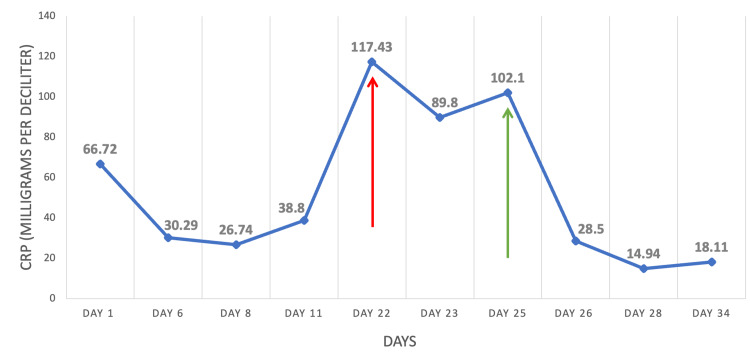
Timeline of CRP level in milligrams per deciliter (mg/dL) Day 1 represents the day of admission. On day 22 (red arrow), CDC was diagnosed and broad antifungal therapy was initiated. On day 25 (green arrow), CDC-induced IRIS was suspected and corticosteroids were added. On day 26, CRP dropped to 28.5 mg/dL, which was 24 hours after corticosteroid initiation. Day 34 showed a CRP of 18.11 mg/dL, which was 72 hours after corticosteroid initiation. CRP: C-reactive protein; IRIS: immune reconstitution inflammatory syndrome; CDC: chronic disseminated candidiasis

Shortly after she was started on corticosteroids, she showed signs of clinical recovery with the resolution of fever and a dramatic decline in inflammatory markers (Figure 3). As a result, the patient was sent home with oral fluconazole for 12 months. On her two-week follow-up appointment, a full resolution of the skin lesions was noted. Moreover, a follow-up abdominal ultrasound after nine months showed no evidence of visceral abscesses or any concerning features for metastatic fungal infection. 

## Discussion

CDC, or hepatosplenic candidiasis, is a disease most commonly affecting patients with hematological malignancies recovering from prolonged neutropenia [1]. It presents as prolonged fever, abdominal pain, and multiple abscesses most commonly in the liver and spleen, and rarely in the lungs and kidneys. IRIS is a condition that occurs during the process of immune recovery. It presents as either the onset of a newly identified infection or the paradoxical worsening of a previously diagnosed opportunistic infection, which may include fungal infections [5]. It’s believed that IRIS is associated with CDC development in patients with hematological malignancies after the initiation of chemotherapy in a condition called CDC-related IRIS [4,6]. 

It is assumed that CDC-related IRIS occurs in two phases. The first phase starts with *Candida* species colonizing the gastrointestinal tract, followed by portal vein invasion, and eventually systemic dissemination during the patient's suppressed immune status. In our case, the most probable cause is the chemotherapy (specifically oral methotrexate) causing mucositis and gastrointestinal damage, allowing the dissemination of *Candida tropicalis* to the systemic circulation. The second phase occurs when the immunity begins to recover, resulting in paradoxical clinical deterioration of the patient, which in our case was characterized by persistent fever and emergence of subcutaneous nodules after the initiation of chemotherapy. This phase is caused by the production of pathogen-specific lymphocytes (CD4+ and CD8+), production of proinflammatory cytokines (e.g., interleukin (IL)-6, IL-1β, tumor necrosis factor-alpha (TNF-α), and interferon‐gamma (IFN‐γ)), as well as an increase in the number of neutrophils and monocytes. The antifungal response rapidly becomes excessive with immune imbalance, causing the formation of granulomas in the tissues containing the pathogen, as seen in our patient who developed multiple abscesses in the spleen and kidneys. This local immune dysregulation promotes the development of IRIS [7].

According to the treatment guidelines, corticosteroids are not recommended to treat CDC [8]. However, many cases have been reported using corticosteroids as an adjuvant therapy to treat CDC-related IRIS in hematological malignancy patients. We summarize them in Table 1. The effect of corticosteroids in resolving symptoms is probably achieved by stimulating the anti-inflammatory cytokines and inhibiting the secretion of proinflammatory ones responsible for the development of IRIS. Our patient showed a rapid resolution of symptoms after treatment with adjuvant corticosteroids, as she was afebrile after 72 hours of corticosteroid use. According to the literature, there is no need to discontinue the chemotherapy cycles in patients with ALL complicated with chronic disseminated candidiasis. Our patient was treated with adjuvant corticosteroids parallel to antifungal medications and chemotherapy; this allowed continued ALL treatment without compromising the hematological outcome of her malignancy. For the antifungal therapy, it’s recommended to be continued for several months (median of 4.3 months) as mentioned in the literature [9]. Although amphotericin-B is the gold standard treatment for severe CDC, its nephrotoxicity with prolonged use is the most common concern in treating CDC patients [10]. This made echinocandins a better option regarding efficacy and toxicity for the treatment of CDC-related IRIS [11]. Our patient received antifungal treatment with caspofungin and was discharged home on fluconazole for 12 months as a long-term antifungal therapy.

**Table 1:** Summary of corticosteroid use in CDC complicated by IRIS in hematological malignancy patients. BCL: B-cell leukemia, ALL: acute lymphocytic leukemia, AML: acute myelocytic leukemia, CLL: chronic lymphocytic leukemia; IRIS: immune reconstitution inflammatory syndrome; CDC: chronic disseminated candidiasis

Reference	Number of patients and age	Disease	Corticosteroid regimen	Clinical outcome
Conter et al., 2007 [12]	17-year-old male	BCL	Oral corticosteroids (1 mg/kg/day)	Fever resolved in 24 hours
Legrand et al., 2008 [4]	6 adults and 4 children	ALL (6), AML (3), CLL (1)	Oral corticosteroids (0.5-0.8 mg/kg for adults, 1 mg/kg for children)	Fever and abdominal pain disappeared a median of 4–5 days
Saint-Faust et al., 2009 [13]	12-year-old male and 8-year-old female	AML and ALL	Prednisolone (1 mg/kg)	Both were well and in remission at time of publication
Chandesris et al., 2010 [14]	75-year-old male	AML	Oral corticosteroids (0.5 mg/kg/day increased to 1 mg/kg/day)	Symptoms resolved
Chaussade, et al. 2012 [15]	5 patients	ALL (3), AML (2)	Mean initial corticosteroid dose 0.6 mg/kg/day (Range 0.3–1)	Four patients alive after 3 years of the condition, and one died of relapsed leukemia
Bayram et al., 2012 [16]	16-month-old boy	ALL	Dexamethasone (0.5 mg/kg)	Fever disappeared after 3 days of corticosteroids
Zajac-Spychala et al., 2016 [17]	4-year-old boy	ALL	Dexamethasone (0.5 mg/kg)	Fever disappeared 3 days after starting steroids
Shkalim-Zemer et al., 2018 [18]	6 children	ALL (3), BCL (1), AML (1), aplastic anemia (1)	Oral prednisolone (2 mg/kg/day) or IV methylprednisolone equivalent in 2 divided doses	Symptoms resolved 1-19 days from the introduction of corticosteroids
Fox et al., 2019 [19]	1 child and 2 adults	ALL, myelodysplastic syndrome, AML	Prednisolone (10 mg twice daily for the pediatric patient), dexamethasone 10 mg/day or prednisolone 30 mg/day for adults	Rapid resolution of fever after starting corticosteroids
Al Yazidi et al., 2023 [20]	5 children	Acute leukemia	Prednisolone (1 mg/kg)	Afebrile 48 hours after starting steroids

## Conclusions

CDC is a serious condition that occurs in patients with hematological malignancy, and it can be complicated by a dysregulated immune response known as IRIS. IRIS occurs during the phase of immune recovery and is characterized by excessive cytokines release and systemic inflammatory response. The addition of corticosteroids as adjuvant therapy to the standard antifungal regimen and the chemotherapy for CDC-induced IRIS in hematological malignancy patients is recommended as the use of corticosteroids rapidly improved the patient’s clinical status and outcomes in our case.

## References

[1] Thaler M, Pastakia B, Shawker TH, O'Leary T, Pizzo PA (1988). Hepatic candidiasis in cancer patients: the evolving picture of the syndrome. Ann Intern Med.

[2] Sallah S, Semelka RC, Wehbie R, Sallah W, Nguyen NP, Vos P (1999). Hepatosplenic candidiasis in patients with acute leukaemia. Br J Haematol.

[3] Murdoch DM, Venter WD, Van Rie A, Feldman C (2007). Immune reconstitution inflammatory syndrome (IRIS): review of common infectious manifestations and treatment options. AIDS Res Ther.

[4] Legrand F, Lecuit M, Dupont B, Bellaton E, Huerre M, Rohrlich PS, Lortholary O (2008). Adjuvant corticosteroid therapy for chronic disseminated candidiasis. Clin Infect Dis.

[5] Cheng VC, Yuen KY, Chan WM, Wong SS, Ma ES, Chan RM (2000). Immunorestitution disease involving the innate and adaptive response. Clin Infect Dis.

[6] Rammaert B, Desjardins A, Lortholary O (2012). New insights into hepatosplenic candidosis, a manifestation of chronic disseminated candidosis. Mycoses.

[7] Candon S, Rammaert B, Foray AP, Moreira B, Gallego Hernanz MP, Chatenoud L, Lortholary O (2020). Chronic disseminated candidiasis during hematological malignancies: an immune reconstitution inflammatory syndrome with expansion of pathogen-specific T helper type 1 cells. J Infect Dis.

[8] Lionakis MS, Kontoyiannis DP (2003). Glucocorticoids and invasive fungal infections. Lancet.

[9] Masood A, Sallah S (2005). Chronic disseminated candidiasis in patients with acute leukemia: emphasis on diagnostic definition and treatment. Leuk Res.

[10] Patel R (1998). Antifungal agents. Part I. Amphotericin B preparations and flucytosine. Mayo Clin Proc.

[11] Grover ND (2010). Echinocandins: a ray of hope in antifungal drug therapy. Indian J Pharmacol.

[12] Conter C, Dupont B, Thiesse P, Bienvenu AL (2007). Persistent fever and hepatosplenic candidiasis, efficiency of a corticoid therapy. J Mycol Med.

[13] Saint-Faust M, Boyer C, Gari-Toussaint M, Deville A, Poiree M, Weintraub M, Sirvent N (2009). Adjuvant corticosteroid therapy in 2 children with hepatosplenic candidiasis-related IRIS. J Pediatr Hematol Oncol.

[14] Chandesris MO, Kelaidi C, Méchaï F (2010). Granulocyte colony stimulating factor-induced exacerbation of fungus-related immune restoration inflammatory syndrome: a case of chronic disseminated candidiasis exacerbation. J Microbiol Immunol Infect.

[15] Chaussade H, Bastides F, Lissandre S (2012). Usefulness of corticosteroid therapy during chronic disseminated candidiasis: case reports and literature review. J Antimicrob Chemother.

[16] Bayram C, Fettah A, Yarali N, Kara A, Azik FM, Tavil B, Tunc B (2012). Adjuvant corticosteroid therapy in hepatosplenic candidiasis-related iris. Mediterr J Hematol Infect Dis.

[17] Zając-Spychała O, Ukielska B, Jończyk-Potoczna K, Konatkowska B, Wachowiak J (2016). Chronic disseminated candidiasis complicated by immune reconstitution inflammatory syndrome in child with acute lymphoblastic leukemia. Case Rep Hematol.

[18] Shkalim-Zemer V, Levi I, Fischer S (2018). Response of symptomatic persistent chronic disseminated candidiasis to corticosteroid therapy in immunosuppressed pediatric patients: case study and review of the literature. Pediatr Infect Dis J.

[19] Fox TA, Halsey R, Pomplun S (2020). Rapid clinical response to adjuvant corticosteroids in chronic disseminated candidiasis complicated by granulomas and persistent fever in acute leukemia patients. Leuk Lymphoma.

[20] Al Yazidi LS, Elsidig N, Wali Y, Nazir H (2023). Chronic disseminated candidiasis in children and the role of corticosteroids therapy. Pediatr Infect Dis J.

